# Seromolecular assess of *Toxoplasma gondii* infection in pregnant women and neonatal umbilical cord blood

**Published:** 2019-01-02

**Authors:** Mohammad Menati Rashno, Shirzad Fallahi, Zahra Arab-Mazar, Hassan Dana

**Affiliations:** 1 Department of Biotechnology, Damghan Branch, Islamic Azad University, Damghan, Iran; 2 Department of Medical Parasitology and Mycology, School of Medicine, Lorestan University of Medical Sciences, Khorramabad, Iran; 3 Infectious Diseases and Tropical Medicine Research Center, Shahid Beheshti University of Medial Sciences, Tehran, Iran

**Keywords:** pregnant women, cord blood, serological methods, molecular methods

## Abstract

Toxoplasmosis is considered as one of the most prevalent human parasitic infections that can be transmitted from mother to the fetus. The onset of toxoplasmosis during pregnancy has clinical complications including spontaneous abortion, preterm labor, stillbirth and fetal abnormalities. The aim of this study was to investigate the prevalence of Toxoplasmosis infection in pregnant women and their infants in Lorestan province, Western Iran. Blood and sera samples were collected from 98 pregnant women and their infants. All collected samples were examined for *Toxoplasma gondii* infection by serological tests (ELISA IgM & IgG) and PCR assay. Among the 98 samples of mother and umbilical cord prevalence of anti-*Toxoplasma* IgG, was 34/98 (34.69 %) and 33/98 (33.67 %), respectively. All pregnant women were negative for, anti-*Toxoplasma* IgM while it was found in 5/98 (5.1 %) of umbilical cords. Based on PCR analysis, *Toxoplasma* infection was detected in 5 (5.1 %) and 7 (7.14 %) of mother and umbilical cords, respectively. Molecular test along with evaluation of IgM (P <0.001) and IgG (P = 0.001) were significantly correlated.

## Introduction

*Toxoplasma* *gondii,* the causative agent of Toxoplasmosis, is the parasitic protozoa that is one of the most important ways for transmission of this parasite, so that 5 out of 1000 seronegative women who infected with *Toxoplasma* *gondii* during pregnancy, transmitted parasite to their fetus (Dubey, 1996[5]; Kravetz, 2013[14]). Congenital toxoplasmosis may be observed in different forms that early diagnosis of the onset of the infection during pregnancy in pregnant women is an important issue in order to protect the mother and the fetus (Nowakowska et al., 2006[17]; Svobodova and Literak, 1998[26]). The most important factor among the various factors affecting the outcome of the fetus is its age at the time of getting infection by mother. The complications of congenital toxoplasmosis have an inverse relationship with fetal age, so that the risk of fetal infection in the first 13 weeks of pregnancy is approximately 15 %, after which the risk of infection increases rapidly. The risk of occurrence of clinical symptoms such as fetal death in the uterus and failure of multiple organs, severe neurological complications, such as mental retardation, microcephaly, retinkooroiditis and hydrocephalus, are more pronounced in the first three months of pregnancy (Paquet et al., 2013[19]; Kieffer and Wallon, 2013[13]; Dunn et al., 1999[6]). To diagnose the infection during or before pregnancy it is necessary to detect IgM, specific anti-*Toxoplasma* IgG antibodies (Jenum and Stray-Pedersen, 1998[12]). Since no comprehensive epidemiological study has been conducted in this group in Lorestan province, the aim of this study was to investigate *toxoplasma* in pregnant women and their infants by serological and molecular methods and screening patients with toxoplasmosis and the risk factors of this disease.

## Material and Methods

### Sampling

**Figure 1:**
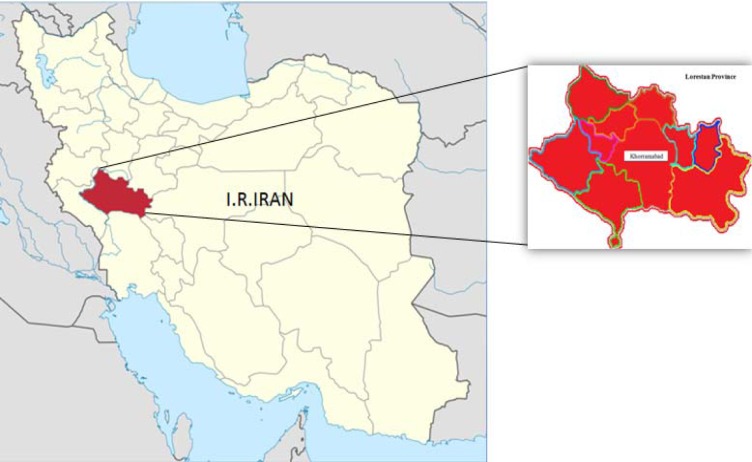
The studied area. Iran map, Lorestan province and Khorramabad city are marked in red

This study is a descriptive cross-sectional type. Our study population included pregnant women and their infants who referred to Asalian Hospital in Khorramabad City in a specific time period (Figure 1(Fig. 1)). In this study, 196 pregnant women and their infants were studied in the second half of 2016. After obtaining written consent from the volunteers, information about them such as age, number of births, and history of abortion was recorded in the questionnaire and blood sample was taken from them. Then their serum and buffy coat were detached at the laboratory of the Faculty of Medicine of Lorestan and kept at -20 °C.

### Serologic test

After completing the sampling, all samples were simultaneously extracted from the freezer and tested according to the instructions of the Kite manufacturer through IgM-ELISA and IgG-ELISA methods (Pioneer of Medicine, Iran).

### Molecular testing

Genomic DNA was extracted from the buffy coats of tissue samples according to the instructions of the manufacturer of the DNA extraction kit, DNG-PLUS (Sina Clone, Iran) and using the molecular technique of PCR, the TOXO1 primer pair (5'GGAACTGCATCCGTTCATGAG 3') and TOXO2 (5'TCTTTAAAGCGTTCG TGGTC 3') prepared for B1 gene which is repeated 35 times in the genome of *Toxoplasma* *gondii* (Rashno et al., 2017[20]) was amplified under the following conditions: initial denaturation of 5 minutes at 94 °C; denaturation of 20 seconds at 94 °C, annealing is 20 seconds at 47 °C, extension 20 seconds at 72 °C, 30 cycles; final extension, 5 minutes at 72 °C. The reaction products are analyzed by electrophoresis in a 1 % agarose gel, which is expected to be a positive PCR reaction product for *Toxoplasma* *gondii* 194 bp.

### Ethical bulletin

This study was approved by the Ethics Committee of Lorestan University of Medical Sciences. Approval number of the Ethics Committee: LUMS.REC. On 4/5/2016, the informed consent was written in this study.

## Results

The gestational age varied between 37 and 40 weeks in the studied mothers. 6 (6.12 %) of the 98 people whose information were registered in this area, had a history of abortion. Among the 98 mothers tested for serum anti-*Toxoplasma* IgG, 34 (34.69 %) positive and 64 (65.30 %) negative samples were detected. Among the 98 serum samples of pregnant mothers tested with ELISA, 34 samples (34.69 %) were evaluated as positive in terms of anti-*Toxoplasma* Specific IgG antibodies. All serum samples of mothers were negative in terms of anti-toxoplasmic IgM antibodies. Among the total 98 umbilical cord blood samples tested by ELISA, 33 samples (33.67 %) were positive in terms of IgG antibodies and 5 (5.1 %) were diagnosed as positive for anti-*Toxoplasma* specific IgM antibodies in their serum samples.

**Figure 2:**
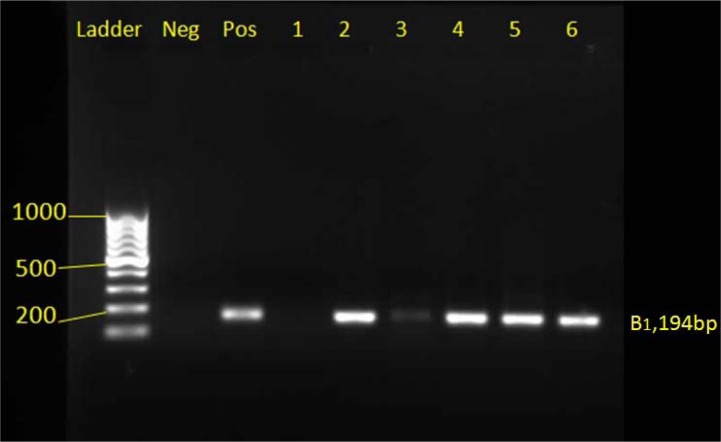
PCR product electrophoresis mothers and babies with toxoplasmosis in Khorramabad city

In the molecular test of PCR, positive infants were detected in 5 samples (5.1 %) of mothers and 7 (7.14 %) neonates using B1 gene on 98 samples of maternal blood and cord blood of their infants (Figure 2(Fig. 2) and Table 1(Tab. 1)).

In this study, Chi-square test was used to evaluate correlation in pregnant women based on the history of abortion (having or without abortion) with serologic ELISA, molecular, and ELISA combined with molecular methods. Among the 6 cases (6.1 %) of pregnant women with abortion history, all had no anti-toxoplasmic IgM antibody, 3 cases (3.1 %) were positive for anti-toxoplasmic IgG antibodies, and the molecular test were evaluated as negative for mentioned women (Table 2(Tab. 2)). Among total of 92 (93.9 %) pregnant women without abortion history, all had no anti-toxoplasmic IgM antibodies, 31 samples (31.6 %) were positive for IgG antibodies, and the molecular test for these women was positive in 3 cases (3.1 %) (Table 2(Tab. 2)). Accordingly, serological ELISA test (P = 0.415) and Molecular test (P = 0.99) have no significant relationship with abortion history in diagnosis of toxoplasmosis.

Studying the correlation between molecular test and serologic ELISA test in pregnant women with positive molecular tests for toxoplasmosis, ELISA test was positive in 7 cases (3.6 %) and 2 cases (1 %) for IgG antibody and IgM antibody, respectively (Table 3(Tab. 3)). In pregnant women with negative molecular tests for toxoplasmosis, ELISA test was positive in 60 samples (30.6 %) and 3 samples (1.5 %) for IgG antibodies and IgM antibodies, respectively (Table 3(Tab. 3)). Accordingly, molecular tests and ELISA test of IgG with an correlation coefficient of 0.123 and P = 0.001 had a significant relationship with each other, the molecular test and ELISA test of IgM with a correlation coefficient of 0.285 and P <0.001 had also a significant relationship together (Table 3(Tab. 3)).

## Discussion

Among human populations, the infection caused by *Toxoplasma* *gondii* has a wide geographical distribution, but its prevalence has been reported to be varied in different regions. So that it is estimated that the prevalence of this parasitic infection in the United States of America and Britain is 16-40 %, and in Central America and the continent of Europe is 50-80 % (Rashno et al., 2017[20]).

Based on the findings of this study, the prevalence of anti-*Toxoplasma* IgG and IgM antibodies was determined using ELISA method in pregnant women referred to the maternity hospital of Asalian Hospital in Khorramabad as 34.69 % and 0 % respectively and in the newborns they were 33.67 % and 5.1 % respectively. The higher serum IgG levels in pregnant women than infants may indicate a higher rate of chronic stage of toxoplasmosis in mothers than infants, which higher serum IgM levels in infants can be related to the weakness of the neonatal immune system and the localization of the *toxoplasma* parasite in the infant's body. Some studies have been conducted in Iran about the prevalence of anti-*Toxoplasma* igG and IgM antibodies in pregnant women using ELISA method. According to this study, the prevalence of IgG was 69.91 % in Amol, 37.8 % in Zanjan, 27.3 % in Khuzestan, 30.8 % in Zahedan, 26.3 % in Tabriz, and 39.8 % in Gorgan, but serum prevalence of igM in pregnant women was 5.9 % in Amol, 1.4 % in Zanjan, Khuzestan and Zahedan, 0.33 % in Tabriz, 3.4 % in Gorgan (Panah et al., 2013[18]; Hajsoleimani et al., 2012[11]; Yad et al., 2014[28]; Ebrahimzadeh et al., 2013[7]; Dalimiasl and Arshad, 2012[3]; Sharbatkhori et al., 2014[24]). The findings of this study are consistent with the findings of other studies in this regard, but in some cases there are obvious differences. So far, the causes of such differences are not fully understood. But factors such as environmental conditions, cultural habits of communities, animal fauna and the level of immunity against parasites are among the factors that can affect the level of infection in an area (Topley et al., 2005[27]).

In previous studies, the prevalence of Toxoplasmosis by serology method in Khorramabad showed that the prevalence of Toxoplasmosis infection in women, residents of the city and the elderly over 60 years was significantly higher than other groups (Rashno et al., 2016[21]). The higher prevalence of *Toxoplasma* in urban areas such as Amol, in comparison to studies conducted in Khorramabad, can indicate the impact of factors such as age, consumption of semi- cooked meat and vegetables on the prevalence of this parasite in the urban population. Climate conditions can also contribute to the transmission of parasite and its prevalence, which has shown in previous studies that areas with rain forests that Amol also are one of these areas can provide long survival for oocytes of the parasite (De la Rosa et al., 1999[4]). There are many studies around the world that indicate a significant relationship between abortion and the prevalence of *toxoplasma*, and a high percentage of abortions caused by toxoplasmosis during pregnancy (Galvan Ramirez et al., 1995[9]; Nissapatorn et al., 2011[16]), the results of our study contradict these reports so that the results of serological and molecular tests did not have a significant relationship with abortion. In this study, in addition to the ELISA serological test, a PCR molecular test was performed to detect *Toxoplasma* *gondii* gene. Based on the results of numerous studies, PCR is a rapid and valid method for the diagnosis of congenital toxoplasmosis (Chabbert et al., 2004[2]; Cermakova et al., 2004[1]; Sławska et al.,2001[25]; Lipka et al., 2001[15]). In toxoplasmosis, IgM antibody is lost after a few months in the patient's serum, but at the onset of a toxoplasmic infection, even in the first days of infection, the PCR method can detect the presence of the parasite, which is a sign of recent infection (Fallahi et al., 2015[8]). In the present study, the molecular method indicates the prevalence of *toxoplasma* in neonates, and in view of the fact that in neonates with IgM positive more molecular samples have been positive indicating the presence of an infection in the neonate blood that results from former research, which indicates the presence and recognition of the DNA of the parasite in people who have positive IgM, can be attributed to the long-term safety of *toxoplasma* (Remington et al., 1968[23][22]). 

In previous studies, 57.9 % of the samples with positive serological results had positive molecular (PCR) results (Guy and Joynson, 1995[10]). This result is greater than the results of our study, and the difference in these results indicates more contamination and *toxoplasma* infection recently, which by more precise molecular methods, the presence of the parasite and its DNA in the blood samples studied. However, the numbers of our study are small, that according to the limited costs and lack of cooperation of pregnant women, we collected less information, and for future research on pregnant women, we suggest studying more samples.

## Conclusion

The results of this study demonstrated high burden of congenital toxoplamosis in this area. A health program is needed to increase the mother's knowledge about toxoplasmosis, and its predictors. Furthermore our results suggested that implementation of newborn screening and follow-up testing can lead to reduce of toxoplasmosis associated complications.

## Acknowledgements

The authors would like to thank the Dr. Mehdi Koushki for their kind cooperation during Research process. Most importantly, the authors would like to thank the participants for volunteering to take part in the study.

## Figures and Tables

**Table 1:**
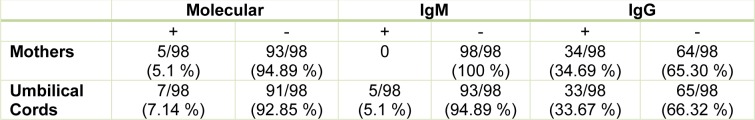
Results from methods performed on maternal and infants blood samples

**Table 2:**
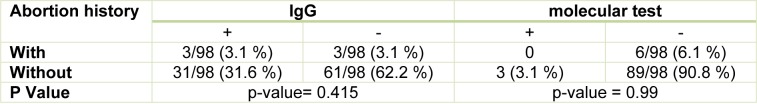
Comparison and correlation between the history of abortion and molecular and serological methods

**Table 3:**
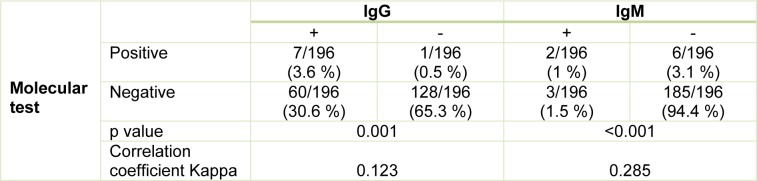
Comparison and correlation of both serological and molecular methods
